# Risk of chemotherapy-related amenorrhoea (CRA) in premenopausal women undergoing chemotherapy for early stage breast cancer

**DOI:** 10.1007/s10549-020-05951-5

**Published:** 2020-10-12

**Authors:** Arran K. Turnbull, Samir Patel, Carlos Martinez-Perez, Anne Rigg, Olga Oikonomidou

**Affiliations:** 1 Cancer Research UK Edinburgh Centre, MRC Institute of Genetics and Molecular Medicine, University of Edinburgh, Edinburgh, UK; 2 Department of Oncology, King’s College Hospital NHS Foundation Trust, London, UK; 3 Department of Oncology, Guy’s and St Thomas’ Hospital NHS Foundation Trust, London, UK

**Keywords:** Chemotherapy, Amenorrhoea, Chemotherapy-related amenorrhoea, Fertility, Premenopausal women, Early breast cancer

## Abstract

**Purpose:**

While chemotherapy has improved survival among younger women with breast cancer, it can induce temporary or permanent chemotherapy-related amenorrhoea (CRA), impacting survival benefit, quality of life and, importantly for younger patients, fertility.

**Methods:**

This single institution retrospective study of 107 premenopausal women with early stage breast cancer who received neoadjuvant or adjuvant combined chemotherapy treatment investigates the association of clinicopathological factors (including age-related, gynaecological and tumour-related variables) with CRA and resumption of menses using generalised linear models for univariable and multivariate analyses.

**Results:**

76% of women developed CRA, of which only 40% resumed menses after treatment. Age at time of treatment and at menarche were significantly associated with CRA incidence, with higher rates linked to older age (≥ 40 years) and later menarche (at ≥ 13 years), in both univariable (*P* = 0.043 and *P* = 0.009, respectively) and multivariate (*P* = 0.010 and *P* = 0.012, respectively) analyses. Age at time of treatment, age at menarche and use of tamoxifen were significantly associated with resumption of menses (with greater resumption rates linked to younger age (< 40 years old), later menarche (≥ 13 years old) or no tamoxifen use status), in both univariable (*P* < 0.0001, *P* = 0.002 and *P* = 0.039, respectively) and multivariate (*P* = 0.001, *P* = 0.011 and *P* = 0.008, respectively) analyses. Menses resumption rates were also significantly higher (*P* = 0.015) in women with later cessation of menses (after 3–6 chemotherapy cycles rather than sooner).

**Conclusions:**

Age at menarche and, specially, at time of treatment are important risk factors for CRA. These variables could aid decision-making for treatment selection and fertility preservation among premenopausal women with early breast cancer.

## Introduction

Breast cancer is the most common invasive cancer diagnosed in women of reproductive age and, indeed, around 25–30% of all breast cancers are diagnosed in premenopausal women [1–3]. Consensus guidelines recommend the use of neoadjuvant or adjuvant chemotherapy for the treatment of this group and studies have demonstrated both a disease-free and overall survival benefit from its use [4, 5]. Although the majority of premenopausal women with early stage breast cancer have an excellent prognosis, the use of neoadjuvant or adjuvant chemotherapy can also induce a number of off-target effects, including temporary or permanent chemotherapy-related amenorrhoea (CRA) [6]. This is thought to result from interference with follicular maturation and potential depletion of the primordial follicles [7–9]. Indeed, the risk of CRA associated with alkylating agents or anthracyclines has been shown to range from 53 to 89% [10, 11]. One study of 78 premenopausal women reported a CRA rate of 44.87% with the FEC regimen (combination therapy with 5-flurouracil, epirubicin and cyclophosphamide) and 23.08% in 66 patients treated with epirubicin plus docetaxel [12]. While some studies have reported a favourable effect from CRA on disease outcome for some premenopausal women, the possible induction of early menopause due to cancer treatment can also have considerable ramifications including fertility issues (of particular importance as more women over the age of 35 consider pregnancy than ever before), sexual dysfunction and, as a result, reduced overall quality of life [6, 13]. While the rate of CRA has been shown to vary with age and chemotherapy dose and regimens administered [3], few studies to date have investigated the importance of other clinicopathological factors. In this single institution retrospective study of 107 premenopausal women with early stage breast cancer, we investigated the association of clinicopathological factors with CRA and the resumption of menses after completion of neoadjuvant or adjuvant combined alkylating agent and anthracycline chemotherapy plus or minus a taxane.

## Material and methods

### Patients

This is a retrospective study of a cohort of patients (*n* = 112) who were treated with chemotherapy (adjuvant or neoadjuvant) for early stage breast cancer (any grade, any histological type, any ER status, HER2 negative only, with or without nodal disease) at King’s College hospital, London, between the years of 2005 and 2010 following appropriate multidisciplinary team discussion. Of the 112 patients, 5 had non-menses prior to the commencement of chemotherapy treatment and were excluded from the analysis, giving a final study cohort of 107 women. All patients had yearly follow-ups for 5 years upon completion of their treatment. Information that was not available at the time through their records, on menarche, menopause, CRA, parity, smoking status and gynaecological history were collected by questionnaire at their yearly follow-up (1–5 year). All patients provided written consent for their clinical data to be reviewed.

### Patient characteristics

At the time of treatment initiation 85% of the patients had regular and 15% had irregular menses. The median age for the cohort was 43 years (range = 35–50 years old) and the median age at menarche was 13 (range = 8–19 years old). The median parity was 2 (range = 0–7) with 54% having had two or more children. At 12 months from chemotherapy completion 16 patients (15%) were premenopausal, 23 (21%) were perimenopausal and 68 (64%) were postmenopausal. Patient characteristics for the cohort of 107 included in the study are summarised in Table 1. 24 patients (22%) reported a previous gynaecological medical history—full details are given in Table 2.

**Table 1:** Patient characteristics and clinical data for the study cohort (*n* = 107)

	Group	Number of patients
Age at treatment	< 40	24 (22%)
	≥ 40	83 (78%)
Age at menarche	< 13	324 (22%)
	≥ 13	83 (78%)
Parity	0	21 (20%)
	≥ 1	86 (80%)
Pre-treatment menses regularity	Regular	91 (85%)
	Irregular	16 (15%)
ER Status	Positive	76 (71%)
	Negative	31 (19%)
Gynaecological history	Yes	25 (23%)
	No	82 (77%)
Smoking status	Smoker	17 (16%)
	Non-smoker	69 (65%)
	Ex-smoker	20 (19%)
Menopausal symptoms at time of data collection	Premenopausal	16 (15%)
	Perimenopausal	23 (21%)
	Postmenopausal	68 (64%)

**Table 2:** Summary of reported previous medical histories of gynaecological conditions in the study cohort (*n* = 107)

Gynaecological history condition	Number of patients
No gynaecological history	83
C-section	1
Colposcopy 2001	1
Endometriosis; CIN 3 & colposcopy	1
Fibroids	3
Fibroids - hysterectomy	1
Fibroids and menorrhagia	2
Fibroids; DUB	1
Fibroids; menorrhagia	1
HIV+	1
Ligation of Fallopian tubes	1
Menorrhagia	1
Menorrhagia; ovarian cystectomy; adenoma	1
POS	5
Sterilised	1
Uterine polypectomy	1
Vaginal prolapse	1
Polypectomy	1

### Treatments

All patients received neoadjuvant or adjuvant chemotherapy. 66 (62%) with node-negative disease received the FEC75 regimen (5-fluorouracil 500 mg/m^2^, epirubicin 75 mg/m^2^, cyclophosphamide 500 mg/m2), in the adjuvant setting and 41 (38%) with node-positive disease received FEC-T (combination therapy including 3 cycles of 5-fluorouracil 500 mg/m^2^, epirubicin 100 mg/m^2^, cyclophosphamide 500 mg/m^2^ followed by 3 cycles of docetaxel 100 mg/m^2^) either neoadjuvantly or in the adjuvant setting). Chemotherapy was given on day 1 every 3 weeks for 6 cycles. All patients completed the treatment schedule. In addition, 72 patients (67%) presented with estrogen receptor alpha (ER)-positive breast cancer at diagnosis and 67 (93%) also received adjuvant tamoxifen after completion of chemotherapy. The five patients who did not receive tamoxifen had contraindications due to previous DVT. All patients included in the study were HER2-negative and therefore none of the above patients received trastuzumab.

### Statistics

The explanatory variables for analysis of CRA were age at time of treatment, age at menarche, parity, pre-treatment regularity of menses, previous gynaecological medical history, ER status of breast cancer (all ER-positive patients received adjuvant tamoxifen), chemotherapy regimen/lymph node status and smoking status. Median, percentiles and range were analysed for each continuous variable. Explanatory variables were analysed using generalised linear models for univariable and multivariate analyses. All statistical analyses were performed in IBM SPSS statistics 25.

## Results

### Incidence of CRA

All of the women in the study (*n* = 107) had menses prior to the commencement of chemotherapy. 91 (85%) had regular menses, while the remaining 16 (15%) reported irregular menses (length of cycle > 35 days or abnormal variation in duration of cycle). Irregular menses prior to treatment was found to be associated with age (*P* = 0.011), with a higher rate recorded in older women. Interestingly, women with irregular menses prior to treatment were also significantly more likely to have ER-positive breast cancer (*P* = 0.038), although only age was retained as significant in multivariate analysis. No other factors were associated with irregular menses prior to treatment.

**Fig. 1:**
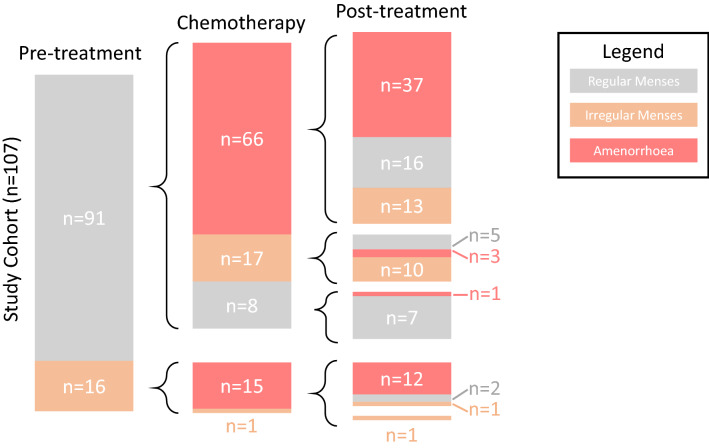
Schematic representing the proportional incidence of CRA and menses resumption following chemotherapy in the study cohort, sub-grouped by regularity of pre-treatment menses

In total, 81 women (76%) developed amenorrhoea during chemotherapy treatment. Of these, 66 (81%) had regular menses prior to treatment and 15 (19%) reported irregular menses. All but one (15/16, 94%) of the women with irregular menses prior to the start of treatment developed amenorrhoea on chemotherapy (Fig. 1). The rates of permanent amenorrhoea were 40.1% in women who reported regular menses prior to treatment and 75% in women who had irregular menses.

Of the 81 women who reported amenorrhoea on chemotherapy, 55 also reported when, during treatment, menses stopped. For the majority of women, menses stopped early in treatment. For 21 women (38% of those who answered this question), menses stopped after a single cycle of chemotherapy, for a further 19 women (35%) menses stopped after 2 cycles and for 12 women (22%) after 3 cycles. Only 3 women (5%) reported that menses stopped during chemotherapy cycles 4 to 6. Only age at time of treatment was found to be significantly associated (*P* = 0.002) with time during treatment when menses stopped. No women under 40 years old reported menses stopping after a single cycle of chemotherapy, but the rate was 44% in women over 40 years old.

Of the 26 women (24%) in the study population who continued menses during chemotherapy, 17 (65%) had regular menses prior to treatment and developed irregular menses during treatment. After completion of chemotherapy 10 of these 17 women resumed regular menses in a period of 4–12 months after treatment completion, while 5 remained irregular and 3 developed post-treatment amenorrhoea. In total, only 7 women in the study cohort (7%), had regular menses prior to chemotherapy and reported no change in menses during or after treatment (Fig. 1).

### Variables associated with CRA

**Fig. 2:**
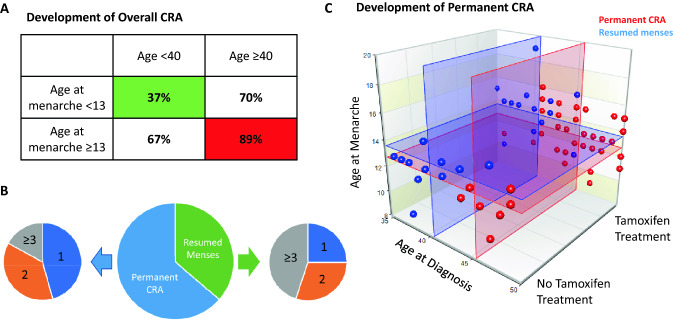
**a** Rates of overall CRA in respect of associated explanatory variables: age at treatment and age at menarche. The highest rates (red) occurred in women aged 40 or older who began menarche aged 13 or older. The lowest rates (green) were found in women aged less than 40 who began menarche before the age of 13. **b** Pie charts showing after which chemotherapy cycle CRA developed in the patients for whom this data was reported (*n* = 55). Comparisons are made between the subgroup of patients who resumed menses after treatment (green) and those who developed permanent CRA (dark blue). Smaller pie charts show the proportions of patients in each subgroup that reported CRA after 1 chemotherapy cycle (blue), 2 cycles (orange) and 3 or more cycles (grey). **c** Scatterplot showing the most significant explanatory variables from the regression analysis. *Horizontal panels* mean age at diagnosis for each subgroup, *vertical panels* mean age at menarche for each subgroup. *Red* subgroup of patients who developed permanent CRA, *blue* subgroup of patients who resumed menses after CRA

Table 3 shows the incidence and rate of CRA in respect of the explanatory variables studied. Age at time of treatment as a continuous variable was found to be significantly associated with incidence of CRA (*P* = 0.043) with significantly higher rates (82%) in women 40 years or older compared with women under 40 (54%) (*P* = 0.008). Age at menarche as a continuous variable was also found to be significantly associated with incidence of CRA (*P* = 0.002) with significantly higher rates (82%) in women who began menarche later (≥ 13 years old) compared with women who began menarche at a younger age (< 13 years old) (54%) (*P* = 0.009). No other explanatory variables were associated with incidence of CRA. There was no direct statistical association (*P* = 0.128) or correlation (R = -0.009) between age at time of treatment and age at menarche and in multivariate generalised linear model analysis both age at treatment (*P* = 0.010, OR 0.262, 95% CI 0.094–0.727) and age at menarche (*P* = 0.012, OR 0.297, 95% CI 0.114–0.770) were significant and contributed to the final model. The rate of CRA was 89% in women 40 years or older who began menarche at 13 years or older. Conversely, the rate in women under 40 who entered menarche before age 13 was 37% (Fig. 2a).

**Table 3:** Association of patient characteristics and clinical data with incidence and rate of CRA and resumption of menses in those with CRA in the study population

	Group	CRA incidence			Incidence of menses resumption following CRA		
		CRA cases per group	Rate of CRA per group	*P*-value	Resumption of menses after CRA per group	Rate of resumption of menses after CRA per group	*P*-value
Age at treatment	< 40	13/24	54%	**0.008**	12/13	92%	**0.002**
	≥ 40	68/83	82%		20/68	29%	
Age at menarche	< 13	13/24	54%	**0.009**	3/13	23%	**0.027**
	≥ 13	68/83	82%		29/68	43%	
Pre-treatment menses regularity	Regular	66/91	73%	0.068	29/66	43%	0.087
	Irregular	15/16	94%		3/15	20%	
Parity	0	17/21	81%	0.531	6/17	35%	0.689
	≥1	64/86	74%		26/64	41%	
ER status	Positive	60/76	79%	0.220	18/60	30%	**0.004**
	Negative	21/31	68%		14/21	67%	
Gynaecological history	Yes	20/25	80%	0.322	9/20	45%	0.562
	No	61/82	74%		23/61	38%	
Smoking status	Smoker	13/17	76%	0.059	6/13	46%	0.593
	Non-smoker	49/70	71%		18/49	37%	
	Ex-smoker	19/20	95%		8/19	42%	
Chemotherapy regimen/lymph node status	FEC-T	50/66	77%	0.986	22/50	44%	0.293
	FEC75	31/41	76%		10/31	32%	

Bold values indicate statistical significance of *P*-value < 0.05

### Resumption of menses following CRA

The overall rate of permanent CRA in the study population was 49.5%. Menses resumed after treatment in only 32 patients of 81 who developed CRA (40%). Of these, 29 women (91%) with regular menses prior to treatment resumed menses after treatment: 16 with regular menses and 13 with irregular menses. One patient who reported irregular menses before treatment resumed irregular menses after treatment. Interestingly, 2 patients with irregular menses prior to treatment resumed regular menses after completion of chemotherapy.

### Variables associated with resumption of menses following CRA

Table 3 shows the rates of menses resumption in those patients who developed CRA in respect of the explanatory variables studied. Age at time of treatment as a continuous variable was highly significantly associated with resumption of menses following CRA (*P* < 0.0001). Women less than 40 years of age were more likely to resume menses after completion of chemotherapy (*P* = 0.002). Age at menarche as a continuous variable was also found to be significantly associated with resumption of menses following CRA (*P* = 0.027) with significantly higher rates of resumption (48%) in women who began menarche later (≥ 13 years old) compared with women who began menarche at a younger age (< 13 years old) (19%) (*P* = 0.013). Tamoxifen treatment was also found to be a significant predictor of menses resumption in univariable analysis (*P* = 0.039). In comparison to tamoxifen-naive cancers, women with ER-positive cancers treated with tamoxifen were less likely to resume menses after treatment. Within the subgroup (*n* = 55) for which time of CRA time of onset data were available, analysis showed that resumption of menses was significantly associated (*P* = 0.015) with later cessation of menses during chemotherapy treatment. In this cohort, a greater proportion of patients (45%) reported development of CRA after cycles 3–6 of chemotherapy in the subgroup of 20 patients (36%) who resumed menses after treatment compared with only 17% in the subgroup of 35 patients (64%) who developed permanent CRA. Conversely, fewer patients report CRA after only 1 cycle of chemotherapy in the group who later resumed menses compared with the group who remained permanently amenorrhoeic (25% vs 46%) (Fig. 2b). In multivariate analysis, age at time of treatment (*P* = 0.001, OR 0.020, 95% CI 0.002–0.022), age at menarche (*P* = 0.011, OR 8.356, 95% CI 1.632–42.788) and tamoxifen treatment (*P* = 0.011, OR 0.184, 95% CI 0.052–0.660) remained significant and contributed to the final model. Age at time of treatment was found to be the most significant factor and overall the rate of permanent CRA in women 40 years and older was 71% compared to 14% in women under 40, irrespective of tamoxifen treatment or age of menarche (Fig. 2c).

## Discussion

### Adjuvant chemotherapy for premenopausal women

Adjuvant chemotherapy has significantly improved the disease-free and overall survival of younger women diagnosed with breast cancer [5, 14]. However, the use of chemotherapy is associated with a number of side effects, due to the non-specific targeting of normal proliferating cells, and these can include gastrointestinal reactions, myelosuppression and suppression or ablation of ovarian function [15, 16]. In this retrospective study we have specifically evaluated CRA in a cohort of women with early stage breast cancer who received adjuvant or neoadjuvant chemotherapy prior to the onset of menopause.

In the study cohort 15% of patients reported irregular menses prior to the commencement of chemotherapy and this was strongly associated with age, with older patients were more likely to have irregular menses. Indeed, this can be attributed to the decreasing number of active ovarian follicles found with increasing age.

### Factors associated with CRA

The rate of CRA in the study group was 76%, which is consistent with previous studies investigating incidence of CRA in premenopausal women receiving alkylating or anthracycline-based chemotherapies [10, 11]. As expected, age was strongly associated with both CRA incidence and development of CRA early in treatment, with women aged over 40 years old more likely to develop CRA early during chemotherapy treatment [12]. Consistent with one study of 431 premenopausal women [17], age at menarche was also strongly associated with CRA. Using a cut-off of 13 years old, the reported UK median age of menarche [18], our results suggest that women who commenced menarche later were more likely to develop CRA. Interestingly, these age-related variables were independent, with no statistical association found between the groups. In multivariate analysis both variables were retained and were found to contribute significantly to the final model. None of the other variables evaluated were found to have a statistical association with development of CRA. Specifically, we compared rates of CRA between patients who received FEC75 with those who received an additional taxane (FEC-T). In accordance with a previous study of 165 premenopausal women [19], the addition of a taxane to the standard FEC75 chemotherapy regimen was not found to increase the rate of CRA.

### Factors associated with resumption of menses after CRA

As expected, resumption of menses was significantly associated with younger age at time of treatment and older age at menarche. In a subset analysis (*n* = 55), resumption was also significantly associated with CRA development later in chemotherapy treatment. In the study population women with tamoxifen-naive breast cancer, the vast majority of which was ER-negative (86%), were also found to be more likely to resume menses compared with those with ER-positive disease who received adjuvant tamoxifen. This is consistent with studies that have shown that tamoxifen can delay the resumption of menses [20]. Overall, age at time of treatment was found to be the most significant explanatory variable in univariable and multivariate analysis. None of the other variables evaluated were found to have a statistical association with resumption of menses.

### Implications of CRA

Some studies have reported that CRA can provide a survival benefit in some groups of women, particularly younger women with ER-positive breast cancer [21–25]. It is thought that this survival advantage results from the indirect endocrine effect of the chemotherapy on ovarian function in addition to its cytotoxic effect. In support of this, the Zoladex Early Breast cancer Research Association (ZEBRA) trial provided evidence for this dual effect. In the trial, premenopausal women were randomised to 2 years of monthly goserelin (gonadotropin releasing hormone (GnRH) superagonist) to block ovarian function or six cycles of chemotherapy. Patients with hormone-sensitive breast cancer were found to have equivalent disease-free survival with either treatment, whereas those with hormone-insensitive disease had improved survival with chemotherapy [26]. That said, more recent evidence suggests that chemotherapy alone is not sufficient for the treatment of younger women with ER-positive breast cancer. One trial demonstrated inferior survival in women younger than 35 years old treated with chemotherapy alone compared with ER-negative disease [27] and another retrospective analysis of 5879 premenopausal women treated with chemotherapy alone reported a significantly higher recurrence rate in women under 35 years old compared with those over [28]. Young women are less likely to develop CRA and it is thought that this inadequate endocrine manipulation accounts for the worse prognosis previously reported in this group with chemotherapy alone [10, 21, 22, 24, 26, 29–34]. Consequently, adjuvant tamoxifen, in addition to chemotherapy, is recommended for the treatment of young women with ER-positive disease. A number of studies have reported that tamoxifen use in addition to chemotherapy increases the rate of CRA and delays the resumption of menses. However, the International Breast Cancer Study Group Trial 13–93, which enrolled 1246 premenopausal women, reported no statistically significant difference in rates of CRA between patients who received tamoxifen and those who did not [3, 21]. Data from the SOFT/TEXT trial suggests that ovarian suppression is superior with GnRH plus exemestane compared with either GnRH plus tamoxifen or tamoxifen alone [35].

Despite the fact that chemotherapy and endocrine therapy have both been shown to improve outcome in younger women with ER-positive breast cancer, there is some debate as to the prognostic relevance of CRA and subsequent resumption of menses after CRA in this group. A meta-analysis of 5513 premenopausal women demonstrated that CRA was a beneficial factor for breast cancer prognosis regardless of whether tamoxifen was administered [36]. One study of 249 women reported that resumption of menses was not associated with poor prognosis [11] while another study of 872 women reported that resumption of menses was an indicator of poor prognosis [37].

CRA also has important implications for fertility and there is a need to address the concerns of younger women who want to remain fertile to have children after a diagnosis of breast cancer [6, 12]. Most women who remain amenorrhoeic 1 year after completion of chemotherapy are unlikely to regain ovarian function, resulting in loss of child-bearing potential [38]. Steps to maintain fertility in women at risk include the use of GnRH agonists, cryopreservation techniques and collection and storage of eggs.

### Study limitations

While the overall findings of this study are consistent with previous studies, the detail described in this study about the likely long-term consequences of timing of cessation of menses during chemotherapy treatment and the later resumption or failure of resumption are not well described in previous studies, and this small retrospective study adds useful data to support clinicians treating such patients. That said, we acknowledge that amenorrhoea is an imperfect surrogate of ovarian failure, however, this was selected as an endpoint as it represents an easily measurable variable that is widely used in the clinical setting. Since we have collected the above data there has been a growing body of evidence supporting the use of anti-Müllerian hormone (AMH) serum levels as a biomarker of ovarian reserve in patients being treated for cancer. AMH levels taken post-treatment may be able to guide advice regarding remaining reproductive lifespan and aid decision-making on suitable adjuvant hormonal treatments such as in hormone receptor-positive breast cancer [39]. Moreover Anderson and Cameron [40] showed that long-term ovarian function after chemotherapy could be predicted by pre-treatment serum AMH concentration. This may also be of value in counselling the patient regarding the need for fertility preservation procedures before commencing therapy [40].

We also acknowledge that due to the retrospective nature of this study the chemotherapy regimen used (FEC75 × 6), with a large cumulative dose of cyclophosphamide, a drug previously reported to be linked to amenorrhoea, is less favoured today. Dose dense chemotherapy regimens are now widely used replacing the previous standard combination regimens of FEC or FEC-T. Platinum agents are also widely used and especially in women keen to preserve fertility docetaxel-carboplatin combination regimens are favoured when possible. There are no data in literature with regards to the effects of these regimens on fertility and ovarian preservation. We have secured funding and obtained ethical approvals to conduct a follow-up study similar in design to the current using though dose dense epirubicin/cyclophosphamide-docetaxel and platinum-based chemotherapy regimens and the results of this retrospective study will be used for comparison purposes. This study will also include pre-treatment and post-treatment measurements of AMH, FSH, LH, oestradiol and patients will be followed up for 5 years with blood collections to measure the above parameters on a yearly basis. Assessing these parameters in pre- and post-chemotherapy longitudinal samples might be also useful in possibly determining the ovarian reserve in women using IUS contraception at the time of diagnosis given that the number of these women has gone up the recent years.

## Conclusions

Given that CRA and subsequent resumption of menses may have important implications for survival benefit, future fertility and quality of life in some groups of patients, it is important to understand which factors are most associated with these phenomena to tailor treatments and take steps to preserve fertility. In this study we have analysed a number of clinicopathological variables and their association with CRA development and resumption of menses. We have confirmed the importance of both age at time of treatment and age at menarche for risk of developing either temporary or permanent CRA. Our data also suggests that age at time of treatment remains the single most important variable for determining the likelihood of developing permanent CRA.
